
JOURNAL INFORMATION
==============================
NLM Title Abbreviation: J Foot Ankle Res
Journal ID: J Foot Ankle Res
NLM Journal ID: 101471610

Journal of Foot and Ankle Research

EISSN: 1757-1146
Publisher: Wiley

ARTICLE INFORMATION
==============================
PMCID: PMC3675418
DOI: 10.1186/1757-1146-6-S1-P16
Article ID: 1757-1146-6-S1-P16
Article version: 1
Subjects: Poster Presentation

Abstract withdrawn

Publication date: 2013
Electronic publication date: 2013 May 31
Volume: 6
Issue: Suppl 1
Supplement: Proceedings of the Australasian Podiatry Council Conference 2013Stefania PenkalaPublication of this supplement was funded by the Australasian Podiatry Council Conference.
First page: P16
Last page: P16
Copyright: Copyright © 2013 licensee BioMed Central Ltd.
Copyright year: 2013
Copyright holder: licensee BioMed Central Ltd.
License: This is an Open Access article distributed under the terms of the Creative Commons Attribution License (http://creativecommons.org/licenses/by/2.0), which permits unrestricted use, distribution, and reproduction in any medium, provided the original work is properly cited.
License URL: https://creativecommons.org/licenses/by/2.0/

Conference date: 2-5 June 2013
Conference name: Australasian Podiatry Council Conference 2013
Conference location: Sydney, Australia

==============================
*Journal of Foot and Ankle Research* 2013, **6**(Suppl 1):P16
http://www.jfootankleres.com/content/6/S1/P16

JOURNAL OF FOOT
AND ANKLE RESEARCH

**POSTER PRESENTATION** **Open Access**

### Abstract withdrawn

*From* Australasian Podiatry Council Conference 2013
Sydney, Australia. 2-5 June 2013

Published: 31 May 2013

doi:10.1186/1757-1146-6-S1-P16
**Cite this article as:** : Abstract withdrawn. *Journal of Foot and Ankle Research* 2013 **6**(Suppl 1):P16.

**Submit your next manuscript to BioMed Central and take full advantage of:**

- **Convenient online submission**
- **Thorough peer review**
- **No space constraints or color figure charges**
- **Immediate publication on acceptance**
- **Inclusion in PubMed, CAS, Scopus and Google Scholar**
- **Research which is freely available for redistribution**

Submit your manuscript at
www.biomedcentral.com/submit

BioMed Central

BioMed Central

© 2013 licensee BioMed Central Ltd. This is an Open Access article distributed under the terms of the Creative Commons Attribution License (http://creativecommons.org/licenses/by/2.0), which permits unrestricted use, distribution, and reproduction in any medium, provided the original work is properly cited.
